# Osteosarcoma of the jaws: a review of literature and a case report on synchronous multicentric osteosarcomas

**DOI:** 10.1186/1477-7819-10-240

**Published:** 2012-11-12

**Authors:** Peter M Nthumba

**Affiliations:** 1 Plastic, Reconstructive and Hand Surgery Unit, AIC Kijabe Hospital, Kijabe, Kenya, 00220, Africa

**Keywords:** Jaw osteosarcomas, Multicentric, Palliative surgery, Synchronous

## Abstract

**Background:**

In the head and neck region, osteosarcoma is the most common primary malignant bone tumor, representing 23% of total head and neck malignancies. Osteosarcomas of the jaws are nevertheless rare lesions, representing only 2 to 10% of all osteosarcomas. This report reviews a single-center histopathology experience with craniofacial osteosarcomas, and reports the management of unusually large synchronous mandibular and maxillary osteosarcomas in a patient.

**Patients and methods:**

A search of the hospital pathology database for specimens with a histological diagnosis of osteosarcomas submitted between July 1992 and May 2011 was made. A chart review of a patient with large synchronous maxillary and mandibular osteosarcomas was performed, and is reported.

**Case presentation:**

A 21-year-old African man with large maxillary and mandibular tumors under palliative care presented with increasing difficulties with eating, speech, and breathing. Surgical debulking was performed, with histology confirming synchronous osteosarcomas of the mandible and maxilla. The patient is well after one year, with no evidence of recurrence, having undergone no further treatment.

**Conclusion:**

Osteosarcomas of the jaw remain enigmatic, and a number of difficulties related to their diagnosis and treatment are yet to be resolved. True synchronous multicentric osteosarcomas of the jaws are extremely rare but, like other osteosarcomas of the jaws, have a favorable outcome, and palliative resection of such lesions, though challenging, can therefore lead to an enormously improved quality of life and self-image, and may even offer the opportunity for cure.

## Background

Osteosarcoma is the most common primary malignant bone tumor, and in the jaws represents up to 23% of total head and neck malignancies [1,2]. Osteosarcomas of the jaws are, however, rare lesions, representing only 2 to 10% of all osteosarcomas [3-5]. Ten percent of these lesions are radiation induced. Hereditary retinoblastoma, Paget’s disease of bone, a history of fibrous dysplasia, or trauma are other factors known to predispose to the development of osteosarcomas [6-9].

Reports on osteosarcoma of the jaws from Africa are few; most reports originate from Nigeria, Kenya, and South Africa [2,3,8,10-12]. Adekeye *et al*. [3] reported finding demographic characteristics in their patient population that were similar to those reported in Western literature. Chindia *et al*. [11] reported on 14 cases of jaw osteosarcomas in patients with a mean age of 30 years (one week to 50 years). The mandible was the most commonly involved bone, as in other studies [1,2,11]. Ogunlewe *et al*. [8] reported that osteosarcoma of the jaw constituted 0.6% of lesions of the jaws and oral cavity over a 21-year period in a Nigerian institution; the patients’ mean age was 27 years. While mandibular lesions occurred predominantly in women, their study suggested a male predisposition of maxillary lesions [8].

Patients with osteosarcomas of the jaw are generally 10 to 20 years older, on average, than those with osteosarcomas of long bones [13]. They have a male to female ratio of 1:1. Studies differ as to the most commonly involved bone [1,2,5,14].

As with a number of other pathologies in sub-Saharan Africa, late presentation of patients with osteosarcoma of the jaws is a common feature [3,8,11]. Thus, the use of adjuvant chemo-radiotherapy with surgical extirpation does not appear to affect outcomes, primarily as the result of late presentation [3,8].

The primary presenting complaints are pain, swelling, paresthesia, and ulceration [5]. Osteosarcomas of the jaws have a different biological behavior from that of osteosarcomas of the long bones, with a lower incidence of metastases and a much better prognosis. This conclusion is based on findings from many case reports and small case series [4,15,16]. Studies on larger cohorts of patients with osteosarcomas of the jaw have suggested a prognosis equivalent to that of long-bone osteosarcomas, which is therefore poorer than previously thought; a conclusion that is not widely accepted at the present time [17,18].

Unusual presentations of osteosarcomas of the jaws, the continuing challenges in establishing both radiological and histological diagnosis, and controversies in treatment and prognosis are the reasons for presenting this case report and literature review.

## Patients and methods

A search of the hospital pathology database for specimens with a histological diagnosis of osteosarcomas submitted between July 1992 and May 2011 was made. A chart review of a patient with large maxillary and mandibular tumors found to be osteosarcomas was drawn up, and the results presented. This study did not require the approval of the hospital ethics committee because it is a retrospective or chart review study.

## Results

A total of 235 specimens submitted between July 1992 and May 2011 to the AIC Kijabe Hospital pathology department were reported histologically as osteosarcomas. Of six patients with osteosarcomas of the craniofacial region, five had jaw tumors. There were two men and three women, with a mean age of 29.5 years. Jaw osteosarcomas represented 2.1% of all osteosarcomas recorded during the study period.

## Case presentation

**Figure 1:**
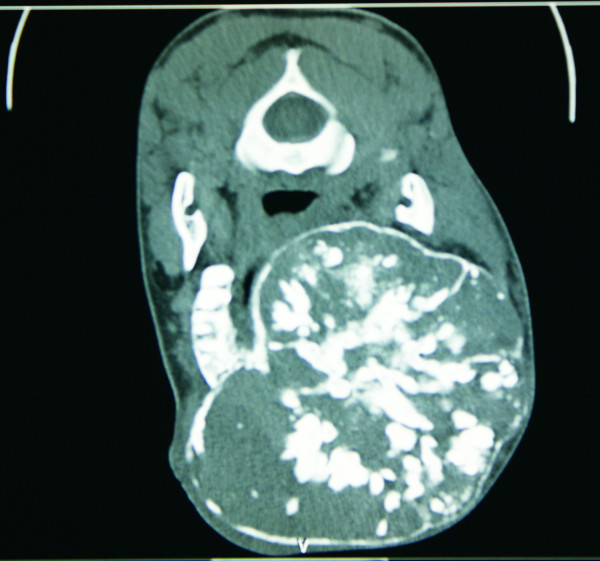
**Photograph of the patient at presentation.** Note the nostrils were completely obstructed by the maxillary tumor.

**Figure 2:**
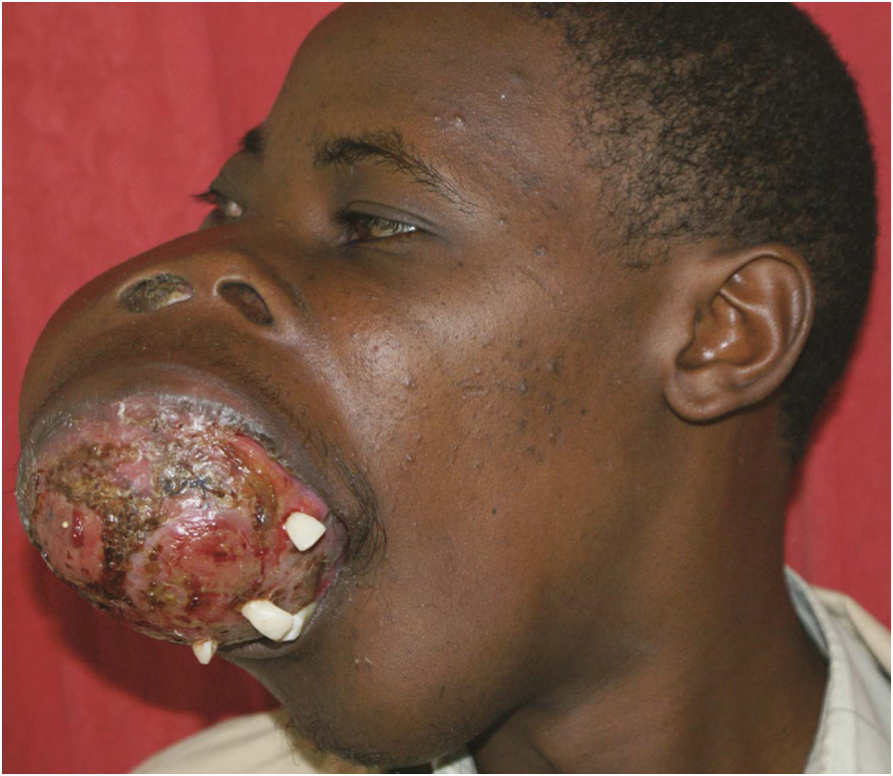
**Lateral (left) photograph, showing an obvious maxillary tumor.** The mouth is maximally open, and had to be used for both feeding and breathing. The patient could only take fluids.

**Figure 3:**
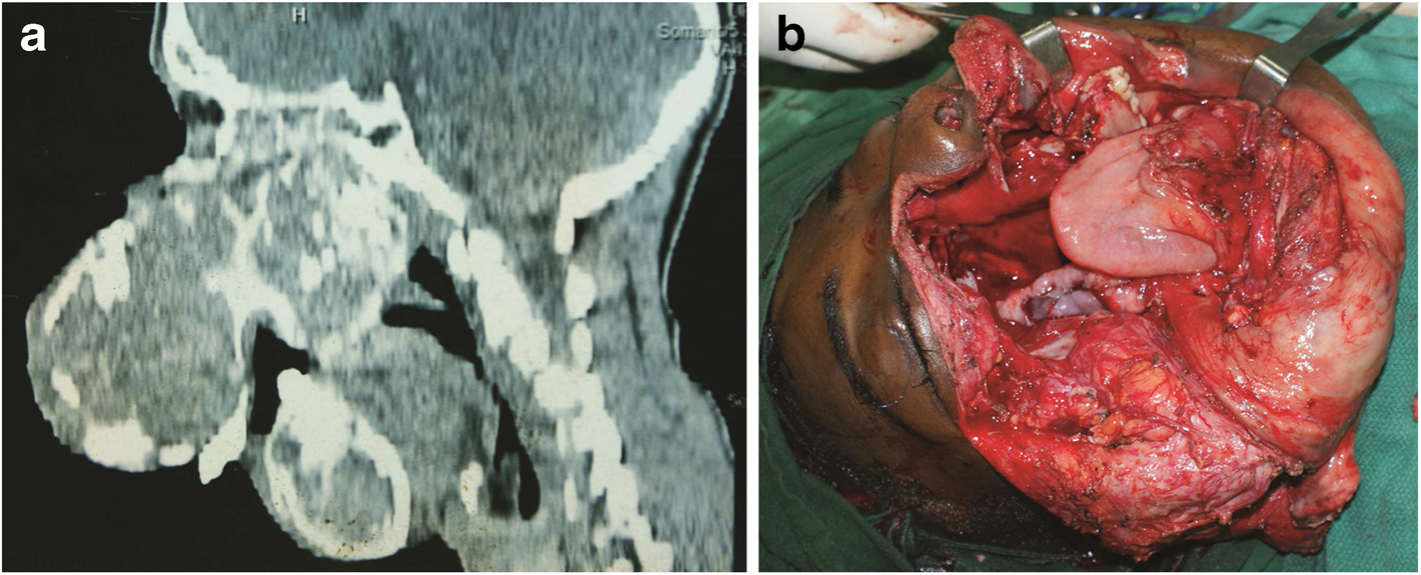
**CT scans.** (**a**) CT scan showing large synchronous maxillary and mandibular tumors. (**b**) Axial CT scan showing extensive maxillary tumor.

A 21-year-old African man presented with a two-year history of facial tumors. He had no other significant medical or family history. He had initially noted a small swelling over his right maxilla, and subsequently felt another in his mandible. The two masses grew over time, even as he sought medical attention. The prohibitive cost of the surgery prevented him from accessing surgical care. At the end of two years, and now with the maxillary and mandibular tumors having grown so large that they interfered with his eating, speech, and breathing, some Good Samaritans offered to help him access medical care in different institutions. A computed tomography (CT) scan and a biopsy were requested at one such institution, and performed (Figures 1, 2, and 3). The CT scan showed two large tumors, one in the right maxilla and the other involving the mandible. A chest radiograph did not reveal any metastases, and there was no evidence of any other lesions. A biopsy of the mandible was reported as suggestive of an osteosarcoma. After discussing the histology, CT scan findings and the patient’s clinical status, the doctors referred the patient for palliative care, at the nearest hospice.

On presentation to the author’s institution, palliative debulking was offered, with the hope of improving the quality of life postoperatively.

**Figure 4:**
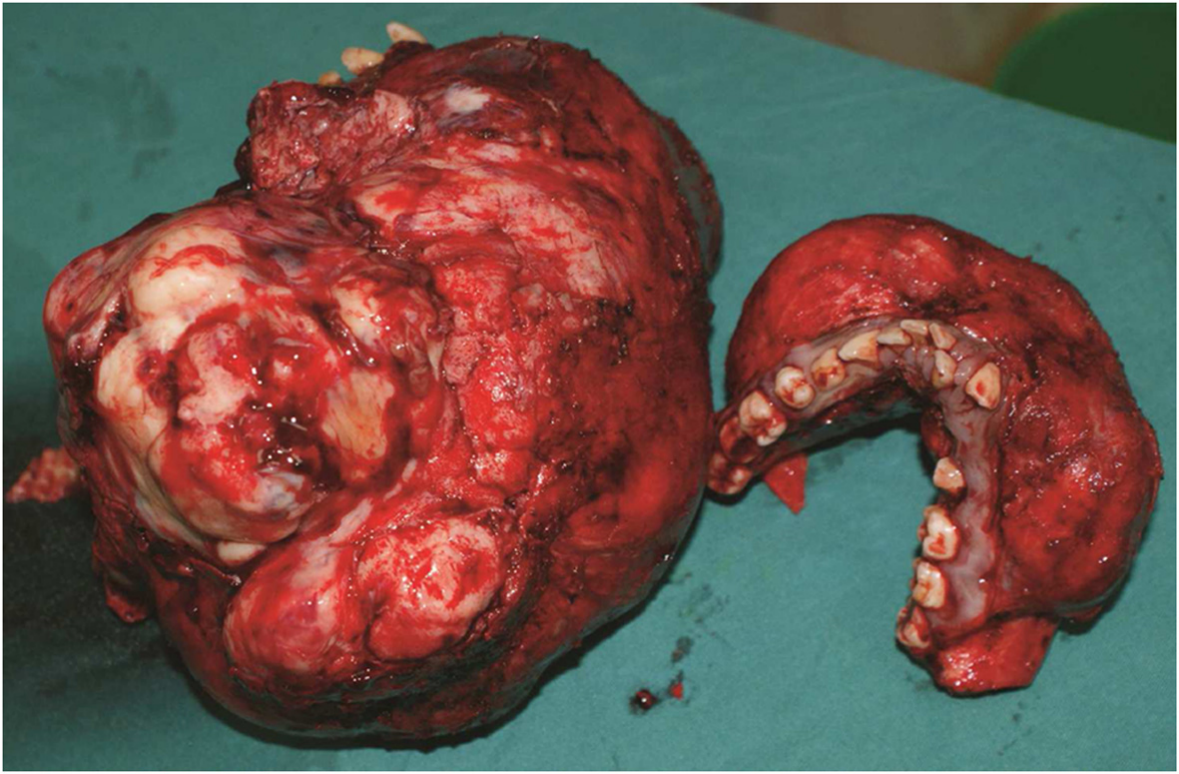
**Surgical procedure.** (**a**) Intra-operative picture showing defect after maxillectomy and mandibulectomy. (**b**) Resected specimens – maxilla and mandible.

**Figure 5:**
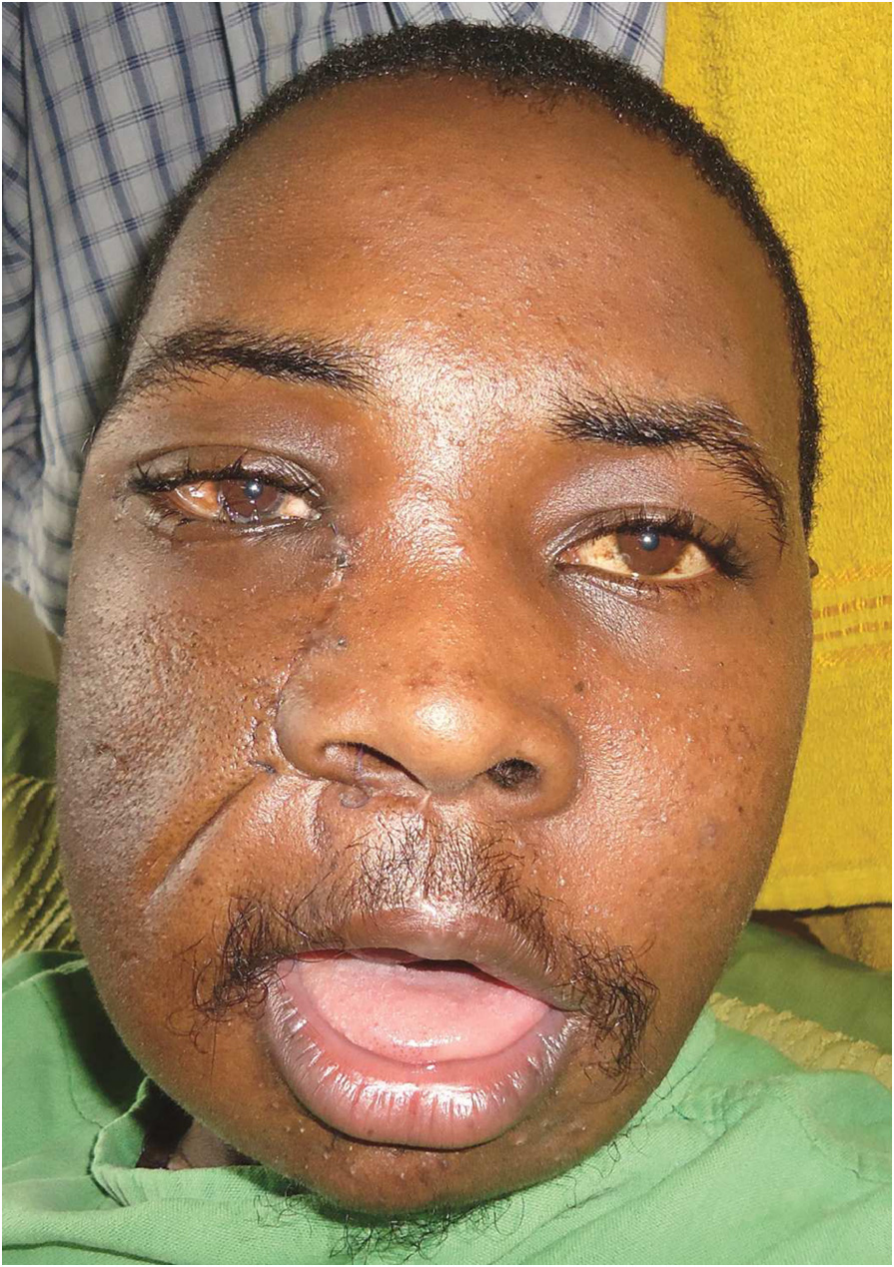
**Excellent cosmetic outcome.** One month postoperatively, the patient could breathe normally, as well as feed on liquids and speak with little difficulty.

At surgery, a right radical maxillectomy and wide excision of the mandibular tumor were performed (Figure 4a). An immediate reconstruction of the palate, nasal lining, and midface with a supraclavicular flap and reconstruction plates was performed, achieving an excellent cosmetic outcome (Figure 5). A feeding tracheostomy and gastrostomy were fashioned to ensure peri-operative ventilation and postoperative nutrition, respectively. The patient’s recovery was uneventful; the tracheostomy tube was removed one week postoperatively, while the gastrostomy feeding tube was removed after three weeks, when it was gaged that the patient was able to feed adequately, orally.

**Figure 6:**
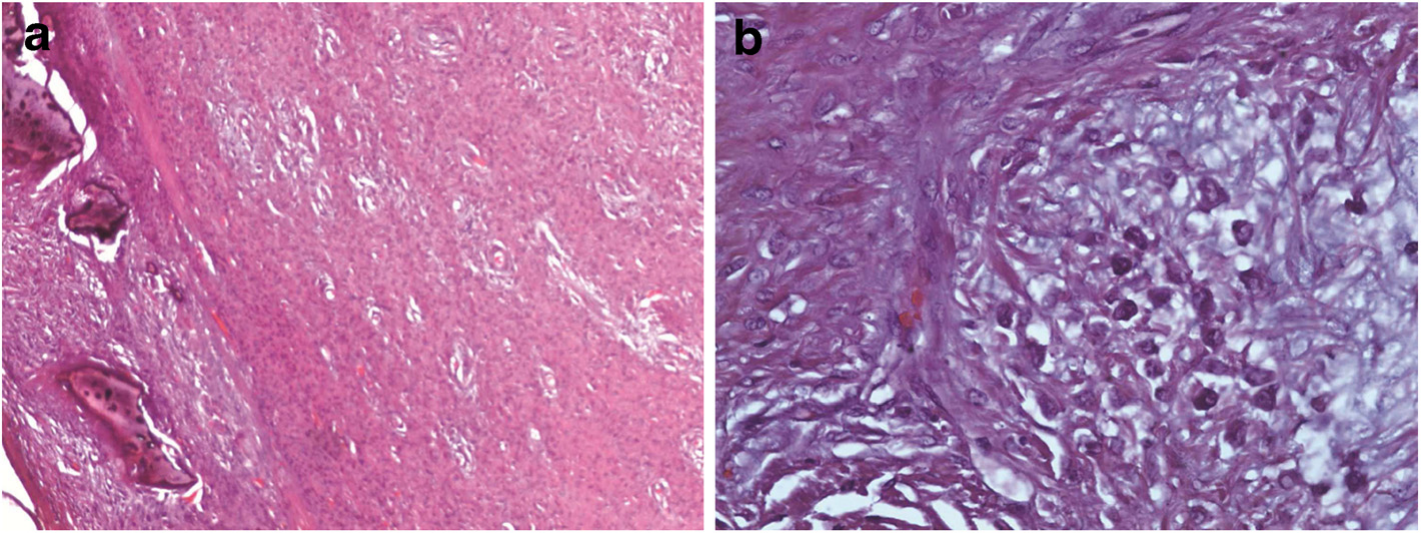
**Hematoxylin and Eosin stains.** (**a**) Magnification 100×. Fibroblastic with tumor high cellularity. Note areas of osteoid formation. (**b**) Magnification 400×. Fibroblastic tumor with high cellularity and nodular myxoid areas. Note moderate nuclear pleomorphism and hyperchromasia.

On histological examination, the mandibular margins were clear, while the maxillary margins were reported as close. The maxillectomy specimen measured 16 cm × 12 cm × 14 cm, while the mandibular tumor was 15 cm × 6 cm (Figure 4b). The histology results were initially delayed, as the pathologists tried to find a diagnosis; a provisional diagnosis of a juvenile ossifying fibroma of both lesions was made, pending further consultation. A final diagnosis of a well-differentiated osteosarcoma of the maxilla and mandible was made by consensus, after a review of the slides (Figure 6a and 6b) and radiographs (Figure 3) by a larger group of pathologists and radiologists.

At the time of discharge from hospital, the patient could feed himself, speak intelligibly, and breathe comfortably. He remains well one year after the operation.

## Discussion

Osteosarcomas of the jaws are challenging lesions in many ways. These tumors are very deforming: they occur in the anatomical region that defines an individual the most, and therefore any deformity here affects the individual in many areas of life. Nothing is more psychologically depressing than rejection by one’s own family, as was the status of the patient presented here. Surgical resection of these tumors is another challenge: adequate resection of these tumors is difficult, especially of the maxilla, because of the complexity of the anatomy, and the resultant defect. Defect reconstruction is a further challenge: facial height and width must be maintained or reconstructed. While the potential for complications in the resection of both the mandible and maxilla is significant, the potential gain even for a purely palliative excision is great, given the cruelty of death from starvation and suffocation that the patient would otherwise have had to face.

The difficulties with histological and radiological diagnosis of osteosarcomas of the jaw are well documented [3,4,16,19-21]. In the case presented here, a provisional diagnosis of juvenile ossifying fibroma was initially entertained, with international consultations leading to a consensus diagnosis of osteosarcoma of both the mandible and the maxilla.

The ‘sunburst’ appearance, the classic radiologic appearance of long-bone osteosarcomas, is not pathognomonic for jaw osteosarcomas. Indeed, the radiologic appearance of jaw osteosarcomas depends on the interplay of three processes: bone formation and mineralization, bone destruction, and periosteal bone formation. Radiologically, the lesions may range from predominantly radiolucent to radio-opaque lesions, with any combinations thereof, depending on the degree of ossification [21]. Plain radiographs and CT scans may be used to define the tumor extent and may thus aid in deciding on resectability, while a chest radiograph or CT scan may be used to detect lung metastasis.

While the initial aim of the surgery was palliative debulking, the resection margins obtained gave hope for the possibility of a cure, especially if adjuvant radiotherapy could be accessed in good time, before any recurrence.

Some authors have suggested that tumor size, location, and histologic grade are the main prognostic factors, although this has been disputed [22], with others stating that tumor size does not appear to be of prognostic value [23]. Early diagnosis and adequate surgical resection are essential for survival, with control of local spread assuring short-term survival. Where adequate resection is possible, surgery alone is adequate, while adjuvant therapy should be considered where surgical margins are inadequate [3,9]. Some authors have suggested that craniofacial osteosarcomas are high-grade tumors, which would suggest a poor outcome in general, but this is not borne out in the general literature, and may be rather an expression of a population-specific variable [7,16]. Only 9% of craniofacial osteosarcomas in a study by Jasnau *et al*. [5] were high-grade tumors.

Daffner *et al*. [24] reported an unusually aggressive osteosarcoma of the mandible, with widespread systemic metastases and death within four months of diagnosis. In contrast, many authors have found most jaw osteosarcomas to be well differentiated [23,25].

Because of easier resectability, and the ability to obtain negative surgical margins, mandibular osteosarcomas have a better prognosis than maxillary tumors [23]. Granowski-LeCornu *et al.*[26] confirmed a poorer outcome for maxillary tumors, related to the difficulty in obtaining adequate resection margins, leading to increased incidence of local recurrence, residual disease, and death. Long-term survival is dependent on such variables as local recurrence, intracranial invasion, and distant spread.

Thus, wide surgical resection forms the most important intervention in the management of osteosarcomas of the jaws, with chemotherapy and irradiation being adjuvant modes, or primary modes in instances of palliation for unresectable lesions [4].

While adjuvant chemotherapy may improve early survival, neither chemotherapy nor radiotherapy appears to impact long-term survival. Because positive surgical margins are associated with poor prognosis, some authors have shown that the use of multimodal therapy may significantly reduce rates of local recurrence, and thereby improve survival [14,18,19,27].

The likelihood of cure in osteosarcomas of the jaws has been estimated at 60% to 70% [7]. Nevertheless, palliative resection, when feasible, even for extensive tumors such as the one reported by the current author, offers the potential for an immediate improvement in the quality of life, including the ability to re-integrate with society, feed, and regain speech and a highly increased degree of self-worth and acceptance.

Multicentric osteosarcomas, defined as lesions present in more than one bone, typically in the absence of pulmonary or other visceral metastases, represent 1 to 2% of all osteosarcomas. They may be synchronous (multiple tumor foci within 5 months of initial presentation) or metachronous (multiple tumor foci 5 months after initial presentation) [24,28,]. Multicentric osteosarcoma cell lines have increased invasiveness and metastatic ability, as well as an affinity for bone; additionally, they have a uniformly poor response to multimodal therapy. Synchronous osteosarcomas (dominant lesions found with smaller synchronous lesions), are associated with a poorer prognosis, because of rapid disease progression and death [28]. The debate as to whether multicentric osteosarcomas represent primarily metastatic disease or multiple primaries is unsettled [28,29]. Another entity found in up to 10% of osteosarcomas is ‘skip’ metastases, where a second focus of osteosarcoma is found in the same bone or in an adjacent bone across a joint [29,30].

It is instructive that, with the exception of a few reports [4,24], almost all the conclusions on the behavior of multicentric osteosarcomas are based on long-bone tumors, which are known to differ significantly in their biological behavior from that of tumors of the jaws, and cannot, therefore, be representative of multicentric osteosarcomas of the jaws [28-30]. Indeed, to date, there are only four reports of multicentric osteosarcomas involving the jawbones in the English literature [31-34].

Behere and Lele [31] reported on a 16-year-old girl found to have a mandibular osteosarcoma and multiple synchronous skeletal lesions. Stroncek *et al*. [32] similarly reported on a 15-year-old girl with mandibular and ilium osteosarcomas, and postmortem pulmonary metastases. They hypothesized that the mandibular tumor was metastatic from the ilium. Ohba *et al*. [33] reported on a 17-year-old girl who presented with mandibular metastasis from an osteosarcoma of the fibula; she subsequently developed systemic metastases. Zhang *et al*. [34] reported on an 18-year-old girl with synchronous osteosarcoma lesions in the mandible and maxilla. Of all the cases of multicentric jaws osteosarcomas in English literature to date, three represent obvious systemic metastatic disease [31-33], with only one [34] involving only the jaws. Interestingly, all the four cases reported involved teenage girls [31-34].

Thus, the current case report represents the first male patient with synchronous jaw osteosarcomas, and the only one from sub-Saharan Africa. The absence of other lesions and the clinical and radiological presentation in the current case lends credence for the synchronous multicentric theory in the evolution of these osteosarcomas. These lesions are most likely to have developed independently in two different anatomical sites, in the same individual. Their size, and the time to surgical excision, is evidence of the different biological behavior exhibited by these tumors, and the better prognosis associated with jaw osteosarcomas when compared with long-bone osteosarcomas. Thus the report by Zhang *et al*. [34] and this report serve as evidence that multicentric jaw osteosarcomas do not necessarily always represent a continuum of metastatic osteosarcoma disease, as proposed by some authors [24,28]. Indeed, one may conclude that the older three reports on multicentric jaw tumors represent purely metastatic disease, from long bones in a disease known to be aggressive, and should not, therefore, be used as evidence of the process of evolution of jaw osteosarcomas [31-33].

Evidence from most studies has shown that jaw osteosarcomas rarely metastasize, lending further weight to the argument that apparently multicentric osteosarcomas involving the jaws and the appendicular skeleton are more likely to be metastatic from the long bones [24,28,31-33].

All authors appreciate the different behavioral patterns of gnathic osteosarcomas compared with long-bone osteosarcomas. It is, therefore, entirely possible that well-differentiated osteosarcomas represent an entirely different entity, one that is related to but is not an actual osteosarcoma. Such an entity would explain the difficulties abounding in the literature on histological and radiological diagnosis, differences in response to treatment, and rarity of metastatic disease with, therefore, a more favorable prognosis of gnathic osteosarcomas [26]. Perhaps the time has come for histopathologists to re-examine diagnostic criteria for jaw osteosarcomas.

## Conclusions

Jaw osteosarcomas remain enigmatic in many ways, and a number of difficulties related to their diagnosis and treatment are yet to be resolved. True synchronous multicentric osteosarcomas of the jaws are extremely rare but, like other osteosarcomas of the jaws, these have a favorable outcome, and palliative resection of such lesions though challenging, can therefore lead to an enormously improved quality of life and self-image, and may even offer the opportunity for cure.

## Consent

Written informed consent was obtained from the patient for the publication of this paper and any accompanying images. A release letter was signed by the patient (thumbprint) permitting publication of features that may lead to his recognition by anyone who knows him, and has been made available to the Editor-in-Chief of this journal.

## Abbreviations

CT: computed tomography.

## Competing interests

The author declares that he has no competing interests. No grants were given for this work, and no financial benefits are expected from this work. This paper has not been presented in any form, in any forum. There is no association between the author with any commercial firm, and no grants were granted for this article. There are no competing interests in the publication of this article.
